# Are elderly people with co-morbidities involved adequately in medical decision making when hospitalised? A cross-sectional survey

**DOI:** 10.1186/1471-2318-11-46

**Published:** 2011-08-18

**Authors:** Anne W Ekdahl, Lars Andersson, Ann-Britt Wiréhn, Maria Friedrichsen

**Affiliations:** 1 Geriatric Department, Vrinnevi Hospital, Gamla Ö vägen 25, 601 82 Norrköping, Sweden; 2 Department of Social and Welfare Studies, Faculty of Health Sciences, Linköping University, Kungsgatan 40, 601 74 Norrköping, Sweden; 3 National Institute for the Study of Ageing and Later Life, NISAL, Linköping University, Kungsgatan 40, 601 74 Norrköping, Sweden; 4 Local Health Care Research and Development Unit, County Council in Östergötland, Linköping University, St. Larsgatan 9 D, 581 85 Linköping, Sweden; 5 Palliative Education and Research Center, Vrinnevi Hospital, Gamla Ö vägen 25, 601 82 Norrköping, Sweden

## Abstract

**Background:**

Medical decision making has long been in focus, but little is known of the preferences and conditions for elderly people with co-morbidities to participate in medical decision making. The main objective of the present study was to investigate the preferred and the actual degree of control, i.e. the role elderly people with co-morbidities wish to assume and actually had with regard to information and participation in medical decision making during their last stay in hospital.

This study was a cross-sectional survey including three Swedish hospitals with acute admittance. The participants were patients aged 75 years and above with three or more diagnoses according to the International Classification of Diseases (ICD-10) and three or more hospitalisations during the last year.

**Methods:**

We used a questionnaire combined with a telephone interview, using the Control Preference Scale to measure each participant's preferred and actual role in medical decision making during their last stay in hospital. Additional questions were asked about barriers to participation in decision making and preferred information seeking role. The results are presented with descriptive statistics with kappa weights.

**Results:**

Of the 297 elderly patients identified, 52.5% responded (n = 156, 46.5% male). Mean age was 83.1 years. Of the respondents, 42 of 153 patients said that they were not asked for their opinion (i.e. no shared decision making). Among the other 111 patients, 49 had their exact preferred level of participation, 37 had less participation than they would have preferred, and 23 had more responsibility than they would have preferred. Kappa statistics showed a moderate agreement between preferred and actual role (κ_w _= 0.57; 95% CI: 0.45-0.69). Most patients wanted to be given more information without having to ask. There was no correlation between age, gender, or education and preferred role. 35% of the patients agreed that they experienced some of the various barriers to decision making that they were asked about: 1) the severity of their illness, 2) doctors with different treatment strategies, 3) difficulty understanding the medical information, and 4) difficulty understanding doctors who did not speak the patient's own language.

**Conclusions:**

Physicians are not fully responsive to patient preferences regarding either the degree of communication or the patient's participation in decision making. Barriers to participation can be a problem, and should be taken into account more often when dealing with hospitalised elderly people.

## Background

Patient participation in medical decision making has long been in focus for many reasons: it is an ethically appealing way to promote patients' status and increase their autonomy [1], it is associated with better treatment results [2-4], and it is stipulated by health care legislation in many countries [5,6]. The literature on the advantages of patient participation is abundant [2,4,7-11], but only sparsely covers participation of older patients [11-13], especially older patients in hospital. The coming half-century will see a dramatic increase in the population of elderly age groups. Due to increased survival to older ages, the prevalence and coexistence of age-related diseases are increasing, leading to co-morbidity. Older patients with co-morbidities are often treated in hospital, and so it is important to increase our knowledge about their conditions and preferences for participation when hospitalised.

To identify elderly people with experience of hospitalisation to answer our questionnaire, we used a definition suggested by the Swedish National Centre of Epidemiology in 2001: "A person over 75 years of age who has been hospitalised three or more times in the last 12 months and has three or more diagnoses in their medical records according to the International Classification of Diseases (ICD-10)" (authors' translation) (SNCE-definition) [14]. These patients account for 13.2% of all hospital inpatient days in Sweden, and consume 19% of all costs of hospital care [15]. In our previous qualitative study of elderly patients with co-morbidities [16], we found quite different attitudes to participation in medical decision making - from a very passive attitude ("whatever you consider best") to a wish for active participation in medical treatment [16]. We also found a desire for more medical information and a hesitation to ask questions about their care. Certain barriers to participation were reported over and over again, for example the severity of the patient's own illness, different doctors with different treatment strategies (to the patient's understanding, not properly communicated between the doctors and/or to the patient), difficulty understanding medical information, and difficulty understanding doctors who spoke the patient's own language with an accent.

It has also been shown in some studies [17,18] that the older the patient, the less desire to play an active role in medical decision making. To our knowledge, however, there have been no studies of the oldest age groups.

The objective of the present study was to investigate the preferred and the actual degree of control, i.e. the role elderly people with co-morbidities wish to assume and actually had with regard to information and participation in medical decision making during their last stay in hospital. How many elderly have experienced one of the following barriers to participation: 1) The severity of the patients' own illness, 2) Different doctors with different treatment strategies, 3) Difficulty understanding medical information, and 4) Difficulty understanding doctors who speak with an accent. Are there any differences regarding the oldest age groups regarding preference for participation in medical decision making?

## Methods

### Study design

We conducted a cross-sectional survey using a questionnaire. Data were collected through telephone interviews. If no contact was established, two more phone calls were made, but no further attempts were made to obtain an answer.

### Setting

The study was performed in a Swedish county with both rural and urban areas and a total of 420 000 inhabitants. There were in total three hospitals with acute admissions: a small district hospital, a hospital in a medium-sized industrial city, and a University Hospital. The patients were identified through the county council's register of care from April 2009 to June 2009. The time between we received the names from the local patient register and the time we attempted to interview was between two weeks and at most, approximately three months.

### Participants

Using the SNCE-definition a consecutive sample of elderly patients was defined and a total of 328 were found. Thirty-one patients without a telephone were excluded, leaving 297 patients to receive a questionnaire. We did not have any additional exclusion criteria, which meant that we attempted to phone even very ill and hospitalised patients. The questionnaire included questions with up to six alternatives, which can be difficult to handle in a telephone interview. To give the patients a chance to read the questions and consider their answers, we posted the questionnaires to them at home 3-7 days before the interview.

### Questionnaires

The intention was to study both the degree of control, i.e. the role that elderly patients with co-morbidities wish to assume in participating in medical decision making during their most recent hospitalisation, and the role they actually had. For this, a modified version of the Control Preference Scale (CPS) was used [19]. The modification consisted of an additional alternative response to the question about actual degree of control in decision making: "I was not asked for my opinion". This modification was found to be important for the aim of the study and was therefore in-corporated. The validated questions from the rest of the Control Preference Scale were also used. The CPS measures the degree of control that an individual wants to assume when decisions are being made about medical treatment and was originally developed as a card sorting technique with pictures describing the different roles the patient and doctor can assume in medical decision making, but today data collection varies. As in several previous studies we chose a telephone interview [10,17,20,21]. The possible responses range from the individual making the treatment decision alone, through the individual making the decision jointly with the physician, to the physician making the decision:

1. I prefer to make the final selection of which treatment I will receive.

2. I prefer to make the final selection after seriously considering my doctor's opinion.

3. I prefer that my doctor and I share responsibility for deciding what treatment is best.

4. I prefer that my doctor make the final decision but consider my opinion.

5. I prefer to leave all decisions regarding treatment to my doctor.

After asking about the preferred role, we asked about the actual role during the most recent hospitalisation, using the same items and modifying the CPS scale with the addition:

6. I was not asked for my opinion.

The response options for the patient's preferred information seeking were:

A. I ask questions about my medical treatment without hesitating.

B. I would like to receive more information about my treatment without having to ask.

C. I find it difficult to ask questions about my treatment.

Again, we also asked about the actual information seeking during the patient's last hospital stay, with the following response alternatives:

A. I asked for information about my medical treatment.

B. I would like to have had more information.

C. I did not want to know about my medical treatment.

To quantify the barriers to participation in medical decision making, we formulated four items based on a qualitative study of a similar patient population [16].

1. Did you feel too ill to be able to take part in the medical decision making?

2. Did you feel that there were too many doctors who were deciding about your treatment?

3. Did you have problems understanding the medical information?

4. Did you have problems understanding things due to doctors speaking Swedish with an accent?

To estimate the importance of these barriers, all the patients who answered "Yes" or "Don't know" to any of the questions about barriers were further asked whether the barrier affected them "a little", "somewhat", or "a lot".

The questionnaire was first tested on six healthy individuals, to ensure that it was usable in a telephone interview. It was then revised and discussed in an expert panel of five, all with more than 15 years of experience in geriatric care and research to get consensus on the formulations of the questions. Finally, the questions were tested on four elderly people with co-morbidities, who proved to be able to answer the questions without difficulty.

If the interviewer suspected that the patient did not understand the questions due to dementia or other illnesses, they asked follow-up questions adapted to the patient's previous answers. Patients were excluded when no understandable answers were received.

### Statistical methods

To evaluate differences between the responding and non-responding groups, we used the Student t-test regarding age and Pearson's chi-square test regarding gender. In the responding group, we further analysed differences in CPS rating and way of information seeking between the categorical variables age, gender, and education, using Pearson's chi-square test. Age was categorised into three age groups of the same size: ≤79, 80-85, and > 85. Student t-tests and Pearson's chi-square tests were performed at a significance level of 5% using version 17.0 of the SPSS software package.

The patients' preferred and actual roles in medical decision making and preferred and actual way of information seeking are presented as percentages, as are their reported barriers to participation in medical decision making.

Discrepancies between preferred and actual roles concerning medical decision making were calculated by subtracting the actual role score from the preferred role score, to give a discrepancy score for each patient centred on 0 (no discrepancy) and ranging from -4 (preferred the most active role but all decisions were made by the doctor) to +4 (preferred the most passive role but the patient had to make the decision on their own). These possible dissimilarities between the preferred and actual role were also analysed as agreement by weighted kappa (κ_w_). The κ_w _value is presented together with a 95% confidence interval (CI). There are no absolute definitions of the interpretation of κ_w_, but the values are usually interpreted as poor if κ_w _< 0.20, fair if κ_w _= 0.21-0.40, moderate if κ_w _= 0.41-0.60, good if κ_w _= 0.61-0.80, and very good if κ_w _= 0.81-1.00 [22].

The patients' preferred and actual way of information seeking and the barriers to information are presented using simple descriptive statistics.

### Ethical considerations

The researchers were not involved in the medical care of the patients. Ethical considerations were observed according to the recommendations of the Helsinki Declaration, as suggested by Wilkie [23]. The study was approved by the Research Ethics Committee at the Faculty of Health Sciences, Linköping University (Dnr M 87-09).

## Results

A total of 297 patients received the questionnaire, and 156 patients chose to participate (52.5%). The reasons for not participating in the study were most often related to the patient being unreachable (15%) followed by not wanting to participate (13%), being too ill to participate (11%), dementia (4%), hearing problems (3%), and being unable to speak Swedish (2%). Being too ill was judged by the patient or a near relative. Being too demented, having hearing problems or being unable to speak Swedish was most often a judgement of the interviewer. The demographics of the respondents are presented in Table 1.

**Table 1:** Demographics of respondents (n = 156)

Mean age (median age)(yrs)	83.1 (83)	
**Range (yrs)**	76-98	
**Gender**		**%**
Male	77	49.4
Female	79	50.6
**Housing**		
Community dwelling	141	90
Special accommodation for the elderly	15	10
**Marital status**		
Married	70	45
Unmarried	26	17
Widowed	60	38
**Education**		
Primary school	99	64
Secondary school	44	28
University	13	8

There were no differences between the respondents and non-respondents regarding age and gender. The mean age was 83.1 and 84.0 years (p = 0.084) and the percentage of women was 44% and 49% (p = 0.33) in the responding and non-responding groups, respectively.

### Preferred and actual roles in decision making

**Figure 1:**
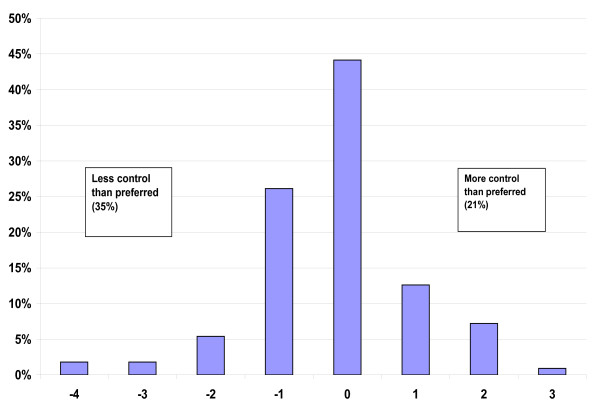
**Discrepancy scores for the preferred versus actual degree of control in medical decision making using the CPS (n = 111)**.

The distribution of the patients' preferred and actual roles in decision making is shown in Table 2. The most common preferred role was for the doctor to make the final decision after considering the patient's opinion (32.7%), and the most common actual role was that the patient was not asked their opinion (27.5%). According to kappa statistics, there was a moderate agreement between the preferred and the actual participation (κ_w _= 0.57; 95% CI: 0.45-0.69). When we excluded the patients describing themselves as "too ill" or "perhaps too ill" to participate in medical decision making, the results remained virtually the same. When calculating the discrepancy score (Figure 1), we excluded the 42 patients who were not asked at all about their opinion in medical decision making during their last hospitalisation. Among the remaining 111 patients, total agreement between preferred and actual participation in medical decision making (a discrepancy score of zero) was seen in 49 patients (44%), while 35% wanted a more active role (negative discrepancy scores) and 21% a more passive role (positive discrepancy scores) than they actually had. In 83% of the patients, the discrepancy score lay between -1 and +1.

**Table 2:** Cross-tabulation showing relationship between preferred and actual degree of control in medical decision making % (n = 153)

	I made the decision about which treatment I received	I made the final decision about my treatment after seriously considering my doctor's opinion	My doctor and I shared responsibility for deciding which treatment was best for me	My doctor made the final decision about which treatment was used, but seriously considered my opinion	I left all decisions regarding treatment to my doctor	I was not asked for my opinion	Total
I prefer to make the final selection of which treatment I will receive	**2.6**	0.0	0.0	0.0	1.3	0.7	4.6
I prefer to make the final selection after seriously considering my doctor's opinion	0.7	**3.9**	4.6	2.6	1.3	6.5	19.6
I prefer that my doctor and I share responsibility for deciding which treatment is best for me	1.3	2.0	**9.8**	6.5	1.3	6.5	27.5
I prefer that my doctor makes the final decision about which treatment will be used, but seriously considers my opinion	0.7	2.6	4.6	**7.2**	7.8	9.8	32.7
I prefer to leave all decisions regarding treatment to my doctor	0.0	0.0	1.3	2.0	**8.5**	3.9	15.7
Total	5.2	8.5	20.3	18.3	20.3	27.5	

### Preferred and actual way of information seeking

About half of the patients preferred to ask questions about their medical treatment, and similarly about half had asked such questions. Almost 40% said they would like to be given more information without having to ask, and 45% would have preferred to have had more information than they did during their last stay in hospital. A very few patients (3%) did not want any information about their medical treatment, while as many as 15% said they found it difficult to ask questions about their treatment.

There were no relationships between age group, gender, and education and the preferred and actual roles in neither decision making, nor any relationships between age group, gender, and education and the preferred or actual roles in information seeking.

### Barriers to patient participation

**Figure 2:**
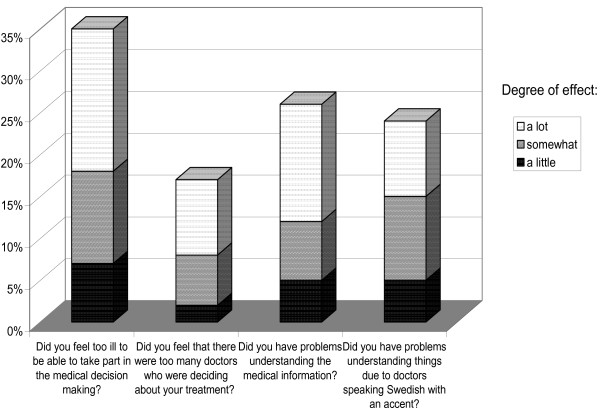
**Frequency of barriers to participation in medical decision making (n = 155)**.

The most common barrier to communication and thus with influence on participation in medical decision making was the patient's own illness, which was noted by 35% of all respondents as an important barrier (Figure 2). The second and third most frequently-reported barriers were difficulty understanding medical information (26%), and difficulty understanding medical information due to linguistic problems (24%).

## Discussion

The results of the present study confirm findings in other patient groups [12,24-27]. Almost half of the patients preferred to play a passive role in decision making, while 35% had *a less active role *than preferred, and 21% *a more active role*. In most studies, being older, being a woman, and being less educated are related to the preference for a more passive role, although this relationship is not constant [18,21,28,29], We did not find any such relationship in our study, just opposite our study showed that generally the patients were less active in medical decision making as preferred. Not finding correlation between education and preferred role in medical decision making could be a cohort effect as elderly is not so well educated as younger in Sweden and the study was too small to show any significances. The findings emphasize the fact that preference for participation is highly individual, and age alone is no excuse for failing to invite the patient to participate in medical decision making.

It is also notable that 27.5% (42 patients) were not asked for their opinion at all. We did not find it statistically appropriate to include this in the calculation of discrepancy scores and kappa statistics. We could assume that the six patients who were not asked about their opinion at all and who preferred to leave all decisions to the doctor were pleased with this outcome, but the remaining 36 patients would have had a discrepancy score between -1 and -4; had we included them, we would have found a poorer agreement between preferred and actual roles than we actually did.

Information and communication are basic requirements for participation [30]. About half of the patients did not feel they were well informed, and some (15%) admitted they were afraid to ask about their health care. The present study emphasizes the wish for information and thus communication, and supports a recent study on acutely admitted elderly patients [31].

In addition to patient participation and provision of information, the questionnaire focused on four negative barriers to communication. The results confirm the importance of all the barriers. The combination of the patient's multifunctional decline (hearing, vision, cognition, etc) and doctors who spoke the patient's language with an accent made it especially difficult to understand medical information. This is an issue that should be noted, and one that has not previously been mentioned much in the medical literature, perhaps because of the risk of sounding xenophobic. Taking into account the increasing opportunities for doctors to migrate, for example, among the European Union countries, perhaps it is a problem that deserves more consideration in the future, particularly in medical specialities where communication is in focus.

One of the limitations in the study was the low response rate; we got answers from only 156 patients of the 297 who received questionnaires. At the same time, it should be remembered that the patients studied were very frail indeed, which was borne out by the fact that as many as 12% had deceased between the time we received their names from the local patient register and the time we attempted to interview them--at most, approximately 3 months. Among the patients who were reported as "unreachable" when we phoned, an unknown number were probably hospitalised. We know from other studies that the mortality rate in this study-population is very high - up to 20-40% per year [32-36].

There was a very low reported prevalence of dementia in our study population (4%) but probably elderly with mild dementia could answer the questionnaire without problems and probably there were a high proportion of patients with moderate or severe dementia among the patients that declined to participate.

The low response rate could be expected because of the high mean age of the patients. A study from Norway of people aged 70 and over showed a rapid decline in response rate from the age group 70-74 (76.6%) successively declining to the age group 95+ where the response rate dropped to 27.1% [37]. We believe that the present study still makes a contribution to knowledge in this field, because to our knowledge it is the first study of participation in medical decision making among this very elderly group.

The CPS scale has been widely used in a variety of populations, ranging from the general public to cancer patients and people with mental disorders [38,39]. Furthermore, it has been found to be clinically relevant, easily administered, valid, and a reliable measure of preferred roles in health care decision making [40,41]. In our study, as in several previous studies, the CPS was used in telephone interviews [10,17,20,21], but face-to-face interviews are always preferable, in order to diminish misunderstandings and get more exact answers.

In the future, it would be of interest to study how doctors perceive the wishes of elderly patients for participation and information. Are doctors aware of the needs but still partly ignore them due to stress or other factors--or are they unaware of the barriers to participation, the different desires for participation, and the demand for information?

## Conclusions

The preferred role in medical decision making is individual and not easy to foresee in elderly (as in younger) patients. This study found no relationships between age group, gender, or education and preferred role. Most elderly people with co-morbidities might not want an active role in medical decision making, but still might want more information about their health care. Our results demonstrate that physicians are not fully responsive to patient preferences regarding the degree of information and participation in decision making.

There are many barriers to communication which affect participation in medical decision making, and it seems worthwhile to consider them when organising hospital care, not least for the elderly patients who make up a large proportion of patients in hospitals. Our point is: "Invite the elderly just as much as younger patients to participation in medical decision making".

## Competing interests

The authors declare that they have no competing interests.

## Authors' contributions

AE drafted the manuscript, LA and MF participated in the design of the study, Anne Ekdahl and A-B W performed the statistical analysis. All authors read and approved the final manuscript.

## Pre-publication history

The pre-publication history for this paper can be accessed here:

http://www.biomedcentral.com/1471-2318/11/46/prepub
